# The Prevalence of Frontal Cells and Their Relation to Frontal Sinusitis: A Radiological Study of the Frontal Recess Area

**DOI:** 10.1155/2013/687582

**Published:** 2013-07-24

**Authors:** Ahmed Z. Eweiss, Hisham S. Khalil

**Affiliations:** ^1^Department of Otolaryngology, Derriford Hospital, Plymouth, UK; ^2^Department of Otolaryngology, Faculty of Medicine, University of Alexandria, Egypt

## Abstract

*Background*. The frontal recess area represents a challenge to ENT surgeons due to its narrow confines and variable anatomy. Several types of cells have been described in this area. The agger nasi cells are the most constant ones. The frontal cells, originally classified by Kuhn into 4 types, have been reported in the literature to exist in 20%–41% of frontal recesses. *Aim of the Study*. To identify the prevalence of frontal recess cells and their relation to frontal sinus disease. *Methods*. Coronal and axial CT scans of paranasal sinuses of 70 patients admitted for functional endoscopic sinus surgery (FESS) were reviewed to identify the agger nasi, frontal cells, and frontal sinus disease. Data was collated for right and left sides separately. *Results*. Of the 140 sides reviewed, 126 (90%) had agger nasi and 110 (78.571%) had frontal cells. 37 frontal sinuses were free of mucosal disease, 48 were partly opacified, and 50 were totally opacified. There was no significant difference found in frontal sinus mucosal disease in presence or absence of frontal cells or agger nasi. *Conclusions*. The current study shows that frontal cells might be underreported in the literature, as the prevalence identified is noticeably higher than previous studies.

## 1. Introduction

Functional endoscopic sinus surgery (FESS) has become one of the commonest surgical procedures performed by otolaryngologists [1]. The widespread adoption of FESS has improved the understanding of the anatomy of the nose and the paranasal sinuses. However, the area which still causes confusion to surgeons is the frontal recess [2]. Surgery in this area is challenging due to its narrow confines and variable anatomy [3]. 

Anatomically, the frontal recess is bounded medially by the middle turbinate and laterally by the lamina papyracea [4]. The posterior wall of the frontal recess is the bulla lamella. If the latter does not reach the skull base, the frontal recess may open into the suprabullar recess. The anterior wall is formed by the frontal process of the maxilla and the frontal bone, which thickens anterosuperiorly to form the frontal beak. In the posteromedial and superior region of the frontal recess lies the lateral wall of the olfactory fossa, which is the thinnest part of the anterior skull base [2]. 

This interesting anatomical area was described by Schaeffer in 1916 as the “nasofrontal region” [5]. However, the first detailed description of the various cells in this area was in 1941 by van Alyea [6], who used the term “frontal recess” rather than “nasofrontal duct.” Van Alyea used the name “frontal cells” in its broader meaning to refer to the different types of ethmoidal cells pneumatizing in this area. This included the frontal cells (sometimes called the frontoethmoidal cells), as described by Kuhn et al. [7], the agger nasi cells, the interfrontal sinus septal cells, and the supraorbital cells. Other cells which have also been described in this area include the suprabullar cells and the frontal bulla cells [8].

The agger nasi is generally considered to be the most constant cell in the frontal recess and was found by Bolger et al. [9] to exist in 98.5% of patients. The term frontal cells (frontoethmoidal cells) is currently used to describe a group of anterior ethmoidal cells that have been classified by Kuhn et al. [7] into 4 types. Type I is a single frontal cell above an agger nasi cell. Type II is a tier of cells in the frontal recess above the agger nasi cell. Type III is a large cell pneumatizing from the frontal recess into the frontal sinus. Type IV is a cell totally isolated within the frontal sinus. Frontal cells have been reported to occur in 20–41% of paranasal sinuses [3]. In our practice, we noticed that reviewing the CT scans of patients admitted for FESS revealed the presence of frontal cells more often than not. Our impression was that the frontal cells might be underreported in the literature.

## 2. Methods

Coronal and axial CT paranasal sinuses scans of 70 consecutive patients admitted for FESS from November 2007 to January 2009 were reviewed as a part of an audit of FESS techniques in our department. The scans were studied to identify the agger nasi and the frontal cells as classified by Kuhn et al. [7]. The cells were identified on the right and left sides separately. Ipsilateral frontal sinus mucosal disease was detected and scored according to the Lund and Mackay system [10]. Other types of frontal recess cells like interfrontal sinus septal cells, supraorbital cells, suprabullar cells, and frontal bulla cells were not included in this study. Fisher's exact test and chi-square test with Yates' correction for tables with 1 degree of freedom were used to test the statistical significance of the difference between frontal sinus disease in presence of agger nasi or frontal cells and frontal sinus disease in the absence of these cells.

As this study was a part of an audit, no approval from the ethics committee was required.

## 3. Results

A total of 140 sides of CT scans of paranasal sinuses were reviewed. Among the 70 patients involved in the study, there were 45 males and 25 females. The age ranged from 18 to 86 years with a mean age of 50.214 (± 16.166).

**Figure 1:**
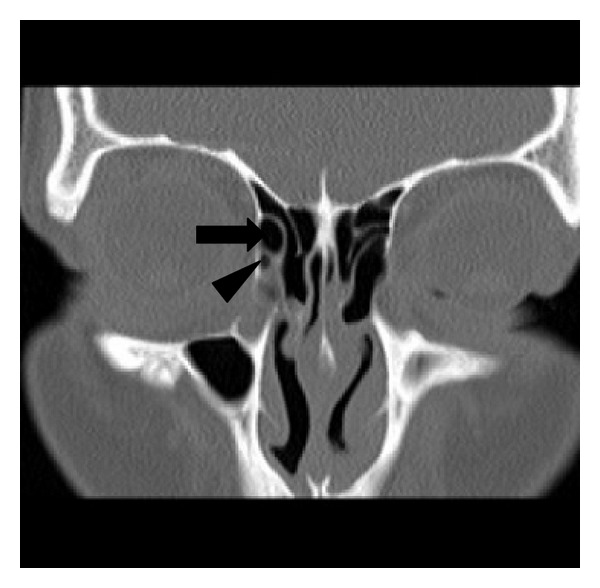
Type I frontal cell and agger nasi cell. A coronal CT scan showing a right-sided type I frontal cell (arrow) above an agger nasi cell (arrow head).

**Figure 2:**
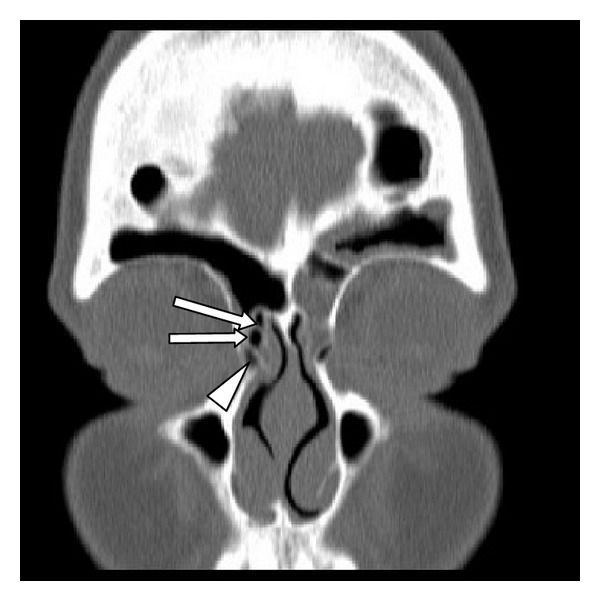
Type II frontal cells and agger nasi cell. A coronal CT scan showing right-sided type II frontal cells (arrows) above an agger nasi cell (arrow head).

**Figure 3:**
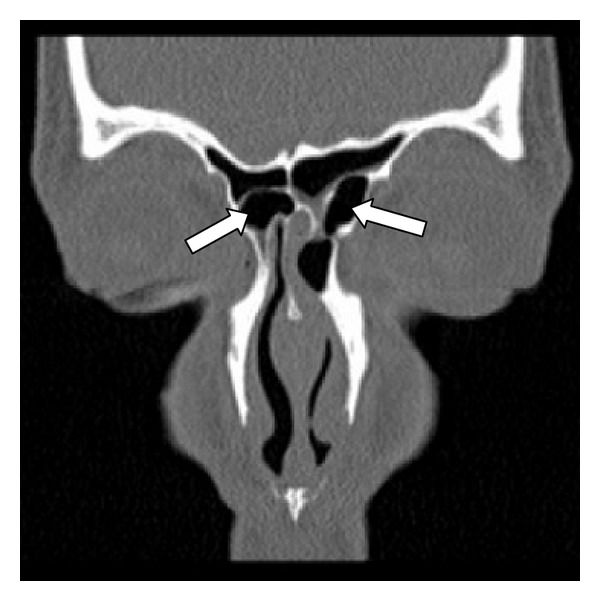
Type III frontal cells. A coronal CT scan showing bilateral type III frontal cells (arrows) pneumatizing from the frontal recesses into the frontal sinuses.

**Figure 4:**
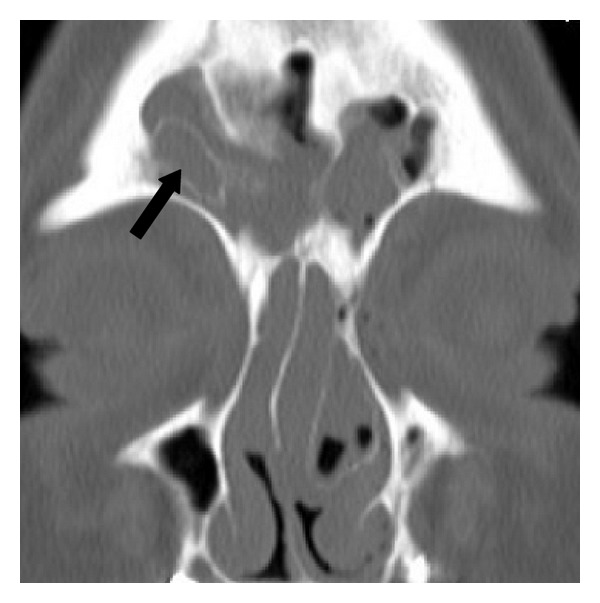
Type IV frontal cell. A coronal CT scan showing a right-sided type IV frontal cell (arrow) lying completely within the frontal sinus.

 Agger nasi cells were found in 126 of the studied sides (90%) (Figures 1 and 2).
 Frontal cells collectively were found in 110 of the studied sides (78.571%) (Figures 1–4).
 Type I frontal cells were found in 30 of the studied sides (21.429%) (Figure 1).
 Type II frontal cells were found in 37 of the studied sides (26.429%) (Figure 2).
 Type III frontal cells were found in 31 of the studied sides (22.143%) (Figure 3).
 Type IV frontal cells were found in 12 of the studied sides (8.571%) (Figure 4).
 Among the studied frontal sinuses, 37 (26.429%) were free from mucosal disease on the CT scans, that is, had a score zero on Lund-Mackay system [10].
 48 frontal sinuses (34.286%) showed partial opacity on the CT scans, that is, had a score 1 on Lund-Mackay system [10].
 50 frontal sinuses (35.714%) showed total opacity on the CT scans, that is, had a score 2 on Lund-Mackay system [10].
 Five frontal sinuses (3.571%) were aplastic or severely hypoplastic and were thus not assessed for mucosal disease.

Frontal sinus mucosal disease was compared in the presence and absence of agger nasi cells, as well as in the presence and absence of frontal cells as a group. Comparison was also made in the presence and absence of each of the 4 types of the frontal cells separately. No significant difference was found between frontal sinus mucosal disease in the presence or absence of any of these cells using both Fisher's exact test and chi-square test with Yates' correction.

## 4. Discussion

In 1941, van Alyea [6] detected frontal cells in 41% of the specimens during cadaveric dissections. He included not only the frontal cells as classified by Kuhn et al. [7], but also the agger nasi, the supraorbital cells, and the interfrontal sinus septal cells. This was most likely an underestimate of the incidence, as many later studies showed the agger nasi on their own to be much commoner than Van Alyea's results. Bolger et al. [9] reviewed more than 200 CT scans and found that the agger nasi cells were present in 98.5% of patients. More recently, Han et al. [11] studied the scans of 202 Chinese subjects free from frontal sinus disease symptoms. They detected the presence of agger nasi in 94.1% of the studied sides. In a study from Poland, Krzeski et al. [12] identified agger nasi cells in 52.87% of the studied sides of CT scans from patients with chronic rhinosinusitis. This reflects the variability in the prevalence of these cells among different studies. In the current study, the agger nasi cells were identified in 90% of the sides of paranasal sinuses CT scans.

Few studies looked into the prevalence of frontal cells, and fewer still investigated the relation between frontal cells and frontal sinus disease. Krzeski et al. [12] identified frontal cells in 23.56% of the studied sides of paranasal sinuses CT scans. Meyer et al. [13] studied the coronal CT scans of paranasal sinuses in a large population. They detected a prevalence of frontal cells in 20.4% of the studied individuals. Their results showed a significantly higher incidence of frontal sinus disease in presence of types III and IV frontal cells. DelGaudio et al. [3] studied the scans of patients presenting for primary and revision sinus surgery. They identified frontal cells on 29.6% of the scans sides in primary patients and 21.9% of the scans sides in revision patients. There was no difference in the frequency of frontal sinusitis in the presence or absence of frontal cells. Finally, Han et al. [11] detected frontal cells in 39.6% of the paranasal sinuses CT scan sides when studying a Chinese population without frontal sinusitis symptoms.

**Figure 5:**
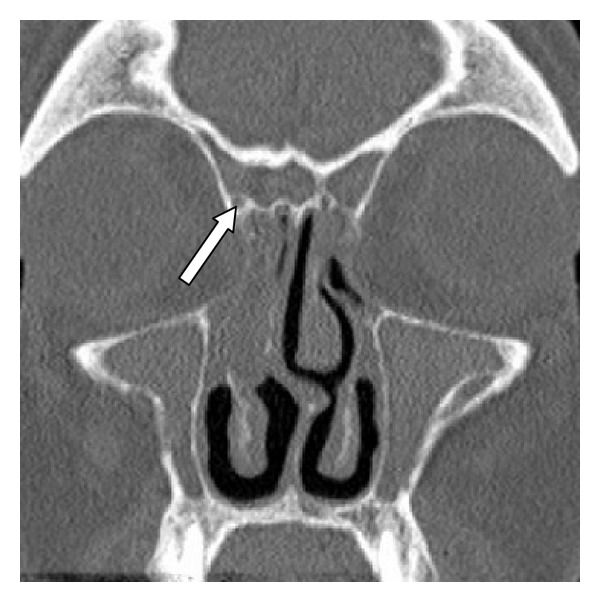
Small type III frontal cell (original figure). A coronal CT scan showing a small right-sided type III frontal cell (arrow) pneumatizing from the frontal recess into the frontal sinus. This cell, although too small to cause frontal sinus obstruction, is still included as a type III frontal cell.

The prevalence of frontal cells in the current study was 78.571%. This is noticeably higher than what was detected by other authors. A possible explanation of this is that we included all the cells that can be named as frontal cells as described by Kuhn et al. [7], regardless of their size. Some of these cells were quite small (Figure 5), and might have been ignored by other authors due to their limited surgical significance. In accordance with DelGaudio et al. [3], the current study showed no significant difference in frontal sinus mucosal disease in presence or absence of frontal cells or agger nasi cells. It can be argued that the patients included in the current study suffered with chronic rhinosinusitis, as they were all patients listed for FESS, and thus the prevalence of the frontal cells in these patients might not represent the prevalence in the general population. However, as the current study and previous studies have shown no relation between the presence of frontal cells and the development of frontal sinusitis, it is likely that the prevalence of frontal cells in patients with chronic rhinosinusitis is not significantly different from the prevalence in a normal population.

## 5. Conclusions

Agger nasi and frontal cells are frequently encountered in the frontal recess area. The results of the current study show that the frontal cells may be underreported in the literature. The presence of these cells does not seem to be significantly related to frontal sinus mucosal disease.
